# PN/PAs-WSe_2_ van der Waals heterostructures for solar cell and photodetector

**DOI:** 10.1038/s41598-020-73152-7

**Published:** 2020-10-14

**Authors:** Xinyi Zheng, Yadong Wei, Kaijuan Pang, Ngeywo Kaner Tolbert, Dalin Kong, Xiaodong Xu, Jianqun Yang, Xingji Li, Weiqi Li

**Affiliations:** 1 School of Physics, Harbin Institute of Technology, Harbin, 150001 China; 2 School of Materials Science and Engineering, Harbin Institute of Technology, Harbin, 150001 China; 3 Collaborative Innovation Center of Extreme Optics, Shanxi University, Taiyuan, 030006 China

**Keywords:** Solar cells, Surfaces, interfaces and thin films, Electronic properties and materials, Two-dimensional materials

## Abstract

By first-principles calculations, we investigate the geometric stability, electronic and optical properties of the type-II PN-WSe_2_ and type-I PAs-WSe_2_ van der Waals heterostructures(vdWH). They are *p*-type semiconductors with indirect band gaps of 1.09 eV and 1.08 eV based on PBE functional respectively. By applying the external gate field, the PAs-WSe_2_ heterostructure would transform to the type-II band alignment from the type-I. With the increasing of magnitude of the electric field, two heterostructures turn into the *n*-type semiconductors and eventually into metal. Especially, PN/PAs-WSe_2_ vdWH are both high refractive index materials at low frequencies and show negative refractive index at high frequencies. Because of the steady absorption in ultraviolet region, the PAs-WSe_2_ heterostructure is a highly sensitive UV detector material with wide spectrum. The type-II PN-WSe_2_ heterostructure possesses giant and broadband absorption in the near-infrared and visible regions, and its solar power conversion efficiency of 13.8% is higher than the reported GaTe–InSe (9.1%), MoS_2_/*p*-Si (5.23%) and organic solar cells (11.7%). It does project PN-WSe_2_ heterostructure a potential for application in excitons-based solar cells.

## Introduction

Since the first fabrication of monolayer graphene by mechanical stripping, two-dimensional (2D) materials have become the focus arising from their depicted excellent electronic and optical properties promising for a wide range of applications^1,2^. Among emerging 2D materials family, black phosphorus (BP) exhibits other attractive properties, such as anisotropic optical properties and the highly tunable and thickness-dependent direct band gap. Despite its many great attractions, a major challenge to the application of BP for electronic and optical applications is its structural instability at ambient conditions. The presence of lone-pair electrons in phosphorene makes it reactive and it can easily absorb impurities with a strong binding energy resulting from the phosphorus to impurity charge transfer. Therefore, enhancing the stability of BP is critically important for device applications. More recently, It has been testified that the group-VA binary compounds 2D materials with the black phosphorene phase such as PN, PAs, AsN and AsSb etc. possess considerable and tunable band gaps, high charge carrier mobilities which exceed the transition metal dichalcogenides (TMDs) materials^3,4^, leading to their favorable application in electronic and energy devices. The PN/PAs show the capability of high solar power conversion efficiency (PCE), pointing to the promising alternative materials for solar cells^3,5,6^. Meanwhile, experimental studies have demonstrated that the PAs has utilization potentiality in the field of mid-infrared (MIR) photoelectron detectors^5–7^. The possibility of widening its electrical performance has also been revealed due to its fully tunable band gap^8,9^. More importantly, group-VA binary compounds have shown good oxidation resist^10^. It has been demonstrated that combining the distinct 2D materials to construct heterostructures can engineer the electronic properties and bring new exciting physical phenomena. For example, the van der Waals heterostructures with type-II band alignment can substantially accelerate spatial separation of photogenerated electron–hole pairs to improve the efficiency of photoconversion. Moreover, it was found that a vertical electric field was able to tune the relative position of the band structure of component monolayers, which enables a controllable Schottky barrier height in the heterostructure. Thus, novel vdW heterostructures that consist of different 2D materials attracted extensive attention due to their unique electronic properties which have abundant opportunities for application in nano-optoelectronics^11,12^.

In this paper, we extended properties of 2D monolayer materials, PN and PAs, by constructing vdW heterostructures with the monolayer WSe_2_ which has great potential application in optoelectronic device due to its considerable and direct band gap and strong photoelectric response^13,14^. Using first principle calculations, the PN-WSe_2_ and PAs-WSe_2_ vdW heterostructures were studied, focusing on their interesting electronic properties enabled by their gate-tunable band gaps and offsets. Their sensitive E-dependent band gaps and wide absorption spectrum offer a practical route to applications in optoelectronics and nano-electronics.

## Calculation details

All calculations have been performed by using the Vienna ab initio simulation package (VASP)^15–17^. Structure optimization have been performed based on the generalized gradient approximation (GGA) within Perdew–Burke–Ernzerhof (PBE) parameterization^18,19^. The Monkhorst–Pack scheme with 9 × 9 × 1 k-point grid was used for the integration in the first Brillouin zone. The lattice parameters and ionic positions were fully relaxed until the total energies and forces were less than 10^−5^ eV and 0.01 eVÅ^−1^, respectively. The DFT-D2 method of Grimme^20^ was used to describe the weak dispersion forces. The vacuum space spanning was set to 40 Å in the z direction. For electronic structures calculation, a 13 × 13 × 1 Monkhorst–Pack mesh was used for both the DOS and band structure. Since PBE functional often underestimates the bandgaps, we have also calculated the band gaps at the HSE06^21^ level for comparison. Optical properties can be described by the dielectric function:$\varepsilon \left( \omega \right) = \varepsilon_{1} \left( \omega \right) + i\varepsilon_{2} \left( \omega \right)$, where $\upvarepsilon _{1} \left( {\upomega } \right)$ is the real part and the $\upvarepsilon _{2} \left( {\upomega } \right)$ is the imaginary part which also represents the dielectric loss. The imaginary part $\upvarepsilon _{2} \left( {\upomega } \right)$ is obtained by calculating the band structure:

$$\varepsilon_{\alpha \beta }^{\left( 2 \right)} \left( \omega \right) = \frac{{4\pi^{2} e^{2} \hbar^{4} }}{{\Omega \omega^{2} m_{e}^{4} }}\mathop {\lim }\limits_{q \to 0} \mathop \sum \limits_{cv} 2w_{k} \delta \left( {\varepsilon_{ck + q} - \varepsilon_{vk} - \hbar \omega } \right) \times u_{ck} |\left( {i\nabla_{\alpha } - k_{\alpha } } \right)|u_{ck} u_{ck} |\left( {i\nabla_{\beta } - k_{\beta } } \right)|u_{ck}$$

while the real part is obtained by through the Kramer–Kronig transformation:

$$\varepsilon_{\alpha \beta }^{\left( 1 \right)} \left( \omega \right) = 1 + \frac{2}{\pi }P\left[ {\mathop \smallint \limits_{0}^{\infty } \frac{{\varepsilon_{\alpha \beta }^{\left( 2 \right)} \left( {\omega^{\prime}} \right)\omega^{\prime}}}{{\omega^{{\prime}{2}} - \omega^{2} }}d\omega^{\prime}} \right].$$

The absorption rate $\alpha \left( \omega \right)$ is

$$\alpha \left( \omega \right) = \sqrt 2 \omega \sqrt {\sqrt {\varepsilon_{1}^{2} \left( \omega \right) + \varepsilon_{2}^{2} \left( \omega \right)} - \varepsilon_{1} \left( \omega \right)}.$$

## Results and discussion

**Figure 1:**
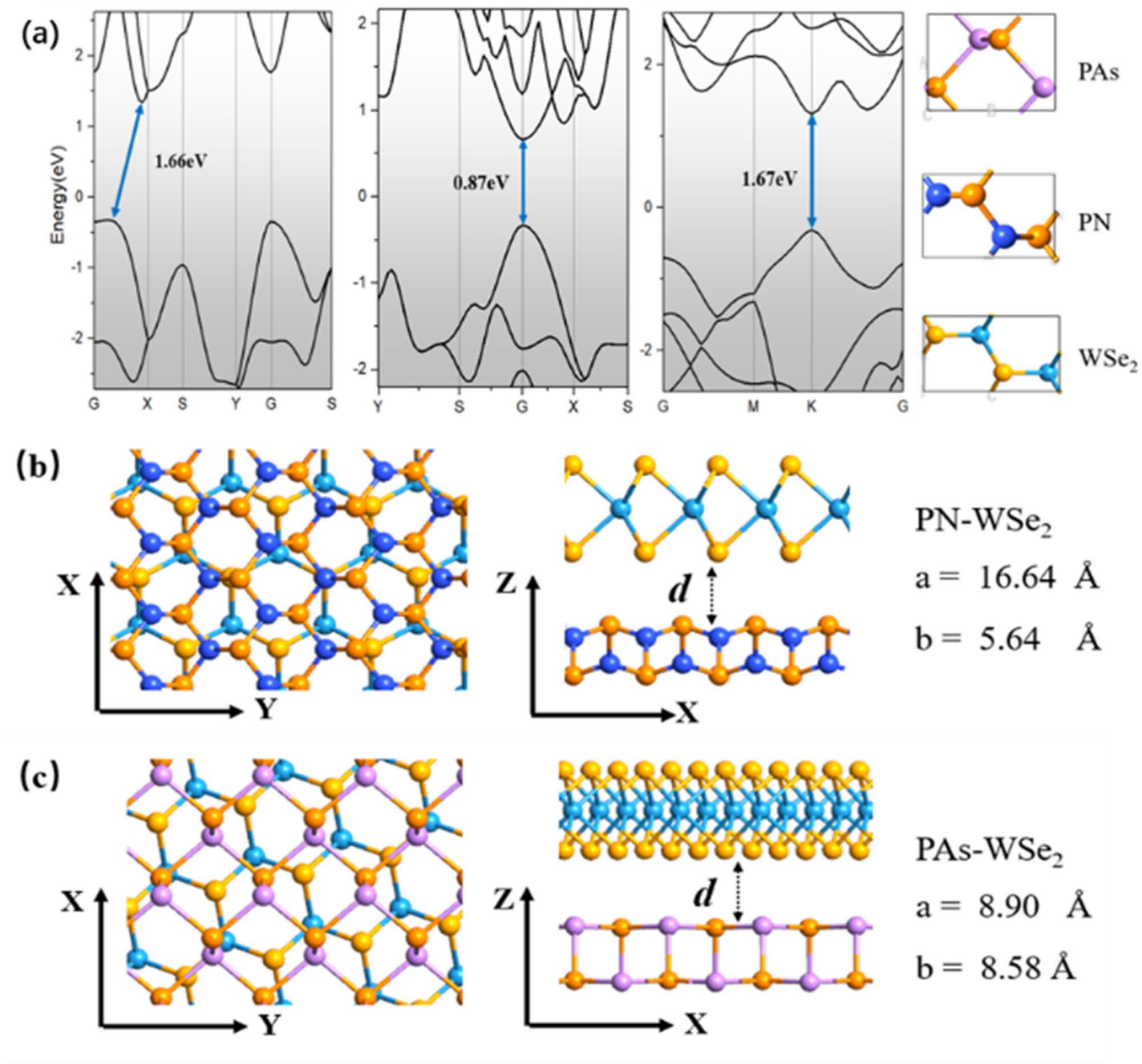
(**a**) The band structures and single optimized rectangular-cell structures of PN, PAs and WSe_2_ are shown; (**b**) The top view, side view and lattice constants of PN-WSe_2_ vdW heterostructure are presented. Deep blue is N, light blue is W, yellow is Se and orange is P. Where the d, the a and the b represent the distance between two monolayers in the heterostructure and lattice constants, respectively; (**c**) The top view, side view and lattice constants of PAs-WSe_2_ vdW heterostructure are presented. Purple is As, light blue is W, yellow is Se and orange is P.

**Figure 2:**
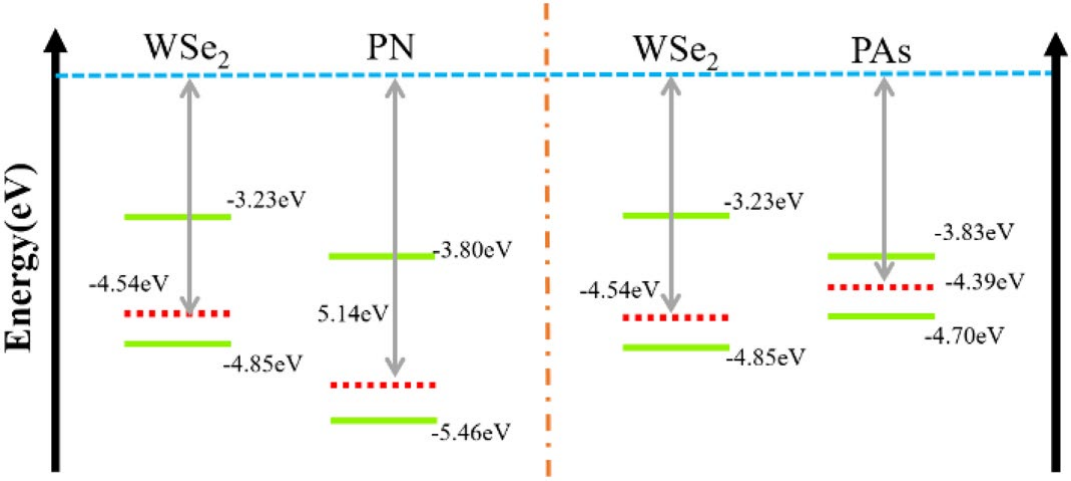
The work function and the position of CBM and VBM relative to the vacuum energy level of WSe_2_, PN, PAs in PBE theoretical level.

**Figure 3:**
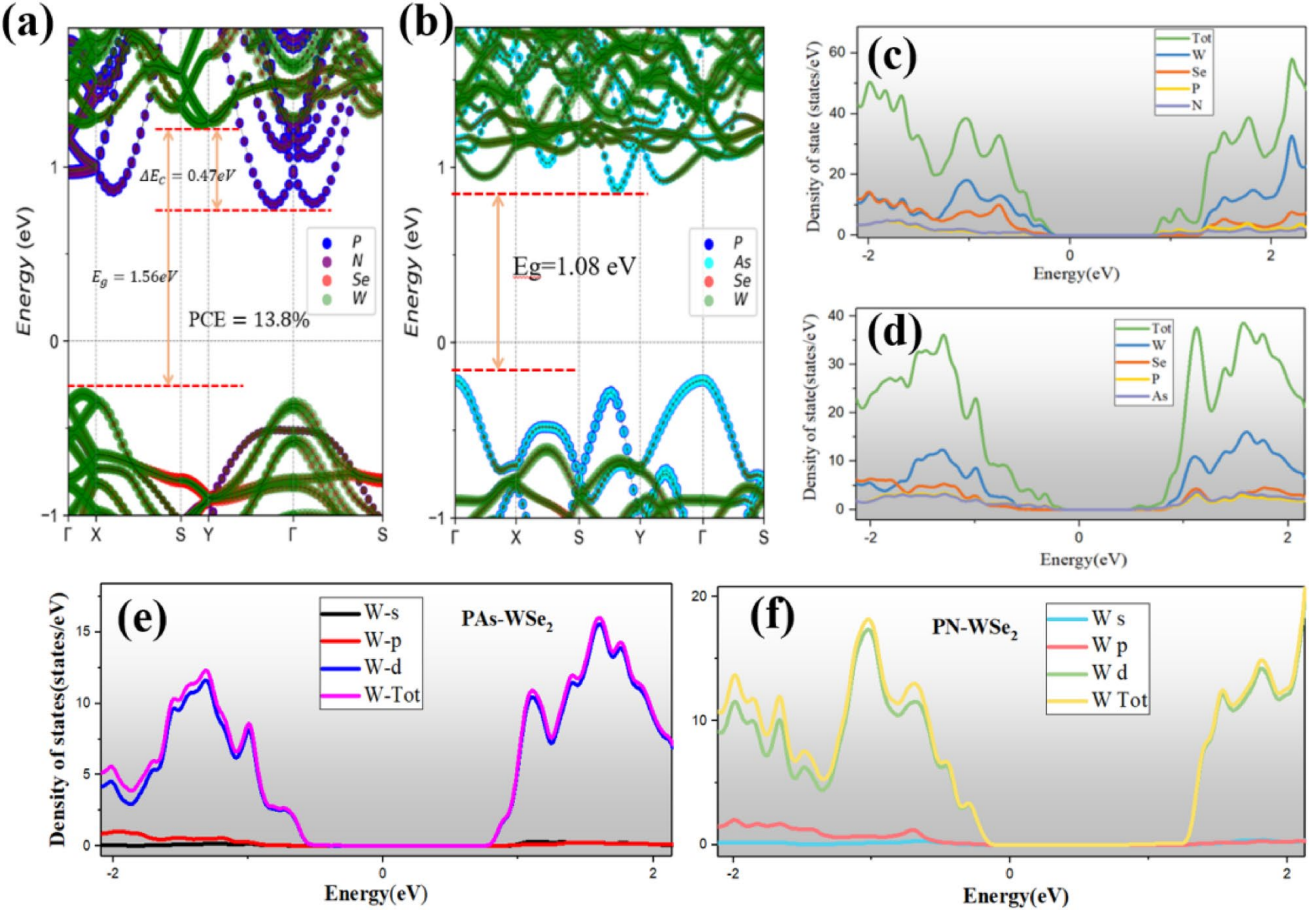
(**a**,**b**) The band structure for PN-WSe_2_ and PAs-WSe_2_ vdW heterostructure; (**c**,**d**) The total and projected DOS of PN-WSe_2_ and PAs-WSe_2_ vdW heterostructure; (**e**,**f**) The contribution from W-orbits for DOS of PAs-WSe_2_ and PN-WSe_2_ vdW heterostructure;

Figure 1 demonstrates the fully optimized rectangular-cell structure of PN-WSe_2_, PAs-WSe_2_ vdW heterostructures and component monolayers with their corresponding band structure. The lattice constants of PN and PAs are a = 2.71 Å, b = 4.15 Å and a = 3.60 Å, b = 4.50 Å, respectively. The lattice constants of rectangular WSe_2_ are a = 3.29 Å, b = 5.70 Å. The optimal interlayer distances of our heterostructures systems are 3.02 Å (PN-WSe_2_) and 3.12 Å (PAs-WSe_2_) through the minimum total energy calculation. These values are smaller than the results of 3.17 Å reported for bP-MoSSe vdW heterostructure^22^. The stability of the two heterostructures determined by the binding energy *E*_*b*_. The formula of the binding energy is:

$$E_{b} = (E - E_{1} - E_{2} )/N$$

where *E* is the total energy of the heterostructure, *E*_1_ is the energy of the isolated PN/PAs monolayer, *E*_2_ is the isolated WSe_2_ monolayer’s energy and *N* is the total number of atoms in the heterostructure. For the stable stack modes with minimum stress presented in Fig. 1b,c, the average stress of both materials is 0.97% (PN-WSe_2_) and 0.82% (PAs-WSe_2_). And the binding energies for PN-WSe_2_ and PAs-WSe_2_ are − 320 meV and − 250 meV, respectively. These values are relatively lower than other possible modes as shown in Table S1 and Table.S2, indicating that WSe_2_ monolayer can combine with the PAs or PN monolayers to form a stable vdWH. As we have known that the band gaps of all structures in GGA-PBE level are lower than HSE06 results. PBE functional predicted that PN monolayer is semiconducting with indirect band gap (Γ-X) of 1.66 eV, whereas PAs monolayer has a direct band gap of 0.87 eV at Γ point. The WSe_2_ monolayer shows a direct band gap of 1.67 eV. Based on HSE06 hybrid functional, band gaps of PN, PAs and WSe_2_ monolayers are 2.72, 1.29 and 2.16 eV respectively. As shown in Fig. 2, the work functions of PN, PAs and WSe_2_ monolayers are 5.14, 4.39 and 4.54 eV on the PBE level. Because their VBM are much closer to the Fermi Level than the CBM, the PN-WSe_2_ and PAs-WSe_2_ heterostructures are both *p*-type semiconductors with 1.09 and 1.08 eV band gaps from the Fig. 3. The similar character is also predicted by using HSE06 hybrid functional. The HSE06 band gaps of PN-WSe_2_ and PAs-WSe_2_ heterostructures are 1.88 and 1.75 eV. The values of work function of PN, PAs and WSe_2_ monolayers are 5.89, 4.92 and 5.12 eV as shown in supplementary information, respectively. In theoretical levels based on PBE and HSE06, the CBM of PAs is lower than that of WSe_2_, whereas its VBM is higher than that of WSe_2_. It indicates that the PAs-WSe_2_ heterostructure is a type-I band alignment heterostructure, while for the PN-WSe_2_ heterostructure, the CBM of PN is lower than that of WSe_2_, whereas the VBM of WSe_2_ is higher than that of PN, indicating that the PN-WSe_2_ heterostructure has a typical type-II band alignment structure.

Figure 3a,b show the projected band structures of two heterostructures based on PBE calculation, respectively. For the PN-WSe_2_ vdW heterostructure, its CBM is contributed by PN monolayer while the VBM is contributed by WSe_2_ monolayer which shows the typical characteristic of type-II band alignment in Fig. 2. It involuntarily separates the electron and hole distributions. Moreover, with projected density of states of vdW heterostructures shown in Fig. 3c,d, it can be seen that the W and Se elements, especially d orbitals of W which is displayed on Fig. 3e,f, have great contribution to the density of states of two heterostructures near the Fermi level. For PAs-WSe_2_ band structure, the type-I band alignment could be testified by its only one band edge contributor PAs which also could be shown in Fig. 2. Despite of some discrepancy in the bandgaps of heterostructures, PBE and HSE06 functionals provide the same band structure and band alignment type. Thus, we only utilize PBE level band structure in following discussion.

**Figure 4:**
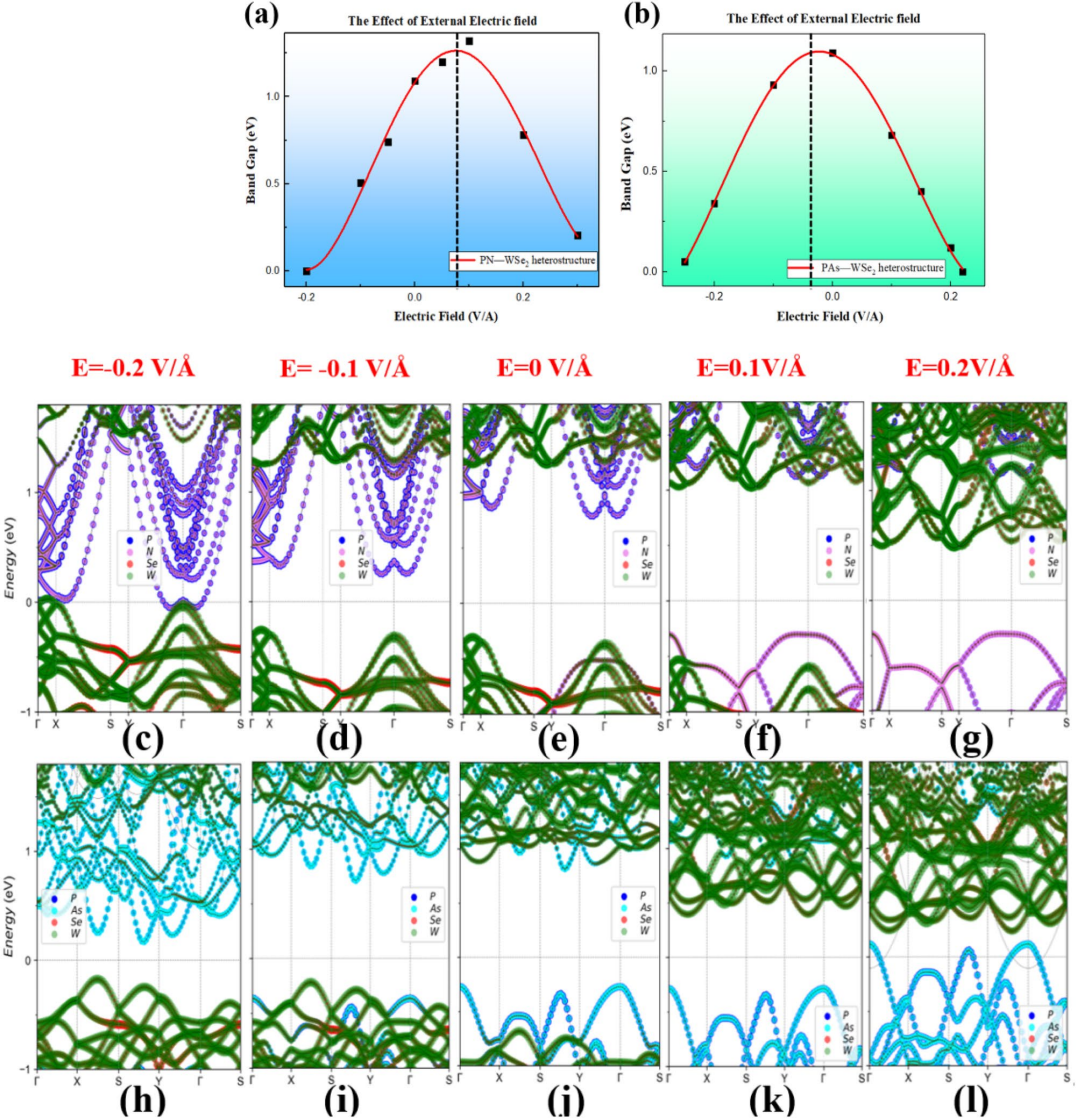
(**a**,**b**) The bandgap modulation by external electrical field, and the band structure under the effect of external electrical field ((**c**–**g**): PN-WSe_2_, (**h**–**l**): PAs-WSe_2_).

In some experiments, the electrostatic gating is an effective way to modulate the electronic characteristics of the vdW heterostructure. By applying the electrical field perpendicular to 2D layers (along *z*-direction) in the range ± 0.25 V/Å, two heterostructures show modulation of band gap. Figure 4a,b demonstrate the fitting curve of the band gap under the electrical field (the correlation coefficients are 0.9834 and 0.9562, respectively). It should be pointed out that variation of band gap shows asymmetric behavior under two opposite electric fields. e.g. The positive field reduces band gap of the PAs-WSe_2_ vdW heterostructure sharply and leads to a transition from semiconductor to metal at E = 0.22 V/Å. The negative field firstly increases the band gap to a maximum value 1.19 eV at E = − 0.025 V/Å and then decreases continuously to minimum 0 eV. On the contrary, for the PN-WSe_2_ vdW heterostructure, it is the negative field that reduces the band gap to 0 eV at E = − 0.20 V/Å, while the positive field make the band gap to a maximum value 1.52 eV at E = 0.075 V/Å and then decreases to 0 eV. It indicated that they possess an inherent electric dipole moment with magnitude 0.075 V/Å and 0.025 V/Å respectively, whose direction depends on materials internal electrostatic potential. Surprisingly, magnitude of electric field causing phase transition of PN/PAs-WSe_2_ heterostructures are much smaller than recently reported BP-MoSe_2_ vdW heterostructure of 1 V/Å^23^ and BP-MoSSe vdW heterostructure of 0.8 V/Å^22^. The projected band structures of the PN-WSe_2_ vdW heterostructures with different electrical field are presented from Fig. 4c–g, while the effect on the band structure of PAs-WSe_2_ vdW heterostructures are shown in Fig. 4h–l. For the PN-WSe_2_ vdW heterostructure, when the external electrical field increases along the positive direction, in contrast to free heterostructure, the PN monolayer has larger contribution to the CBM whereas contribution of WSe_2_ in VBM increases with the band edge position approaching each other as shown in Fig. 4e–g. For the PAs-WSe_2_ vdW heterostructure, when the electrical field along the positive direction increases, contributions of PAs to the CBM increase whereas leading to more contributions from WSe_2_ to the VBM as shown in Fig. 4j–l. Under the negative electric field, we can see the different effect as shown in Fig. 4h,i. When the magnitude of the applied electric field reaches 0.1 V/Å, WSe_2_ has large contribution to both VBM and CBM. It suggests that the PAs-WSe_2_ heterostructure will transform from type-I to type-II band alignment by the electric field which could be used in high-performance optoelectronic nanodevices. In addition, we found the PN-WSe_2_ vdW heterostructure would transform to *n*-type semiconductor from *p*-type semiconductor if the external electric field is large enough regardless of direction. However for the PAs-WSe_2_ vdW heterostructure, only increasing the electrical field in negative direction could induce a transition from *p*-type semiconductor to *n*-type semiconductor. The asymmetric behavior of the band gap modulated by the electrical field can be attributed to the balance between the external electrical field and the internal electrical field of the heterostructures, which are caused by the uneven distribution of charge densities inside the heterostructures when two different components are stacked together by van der Waals interaction^24^.

**Figure 5:**
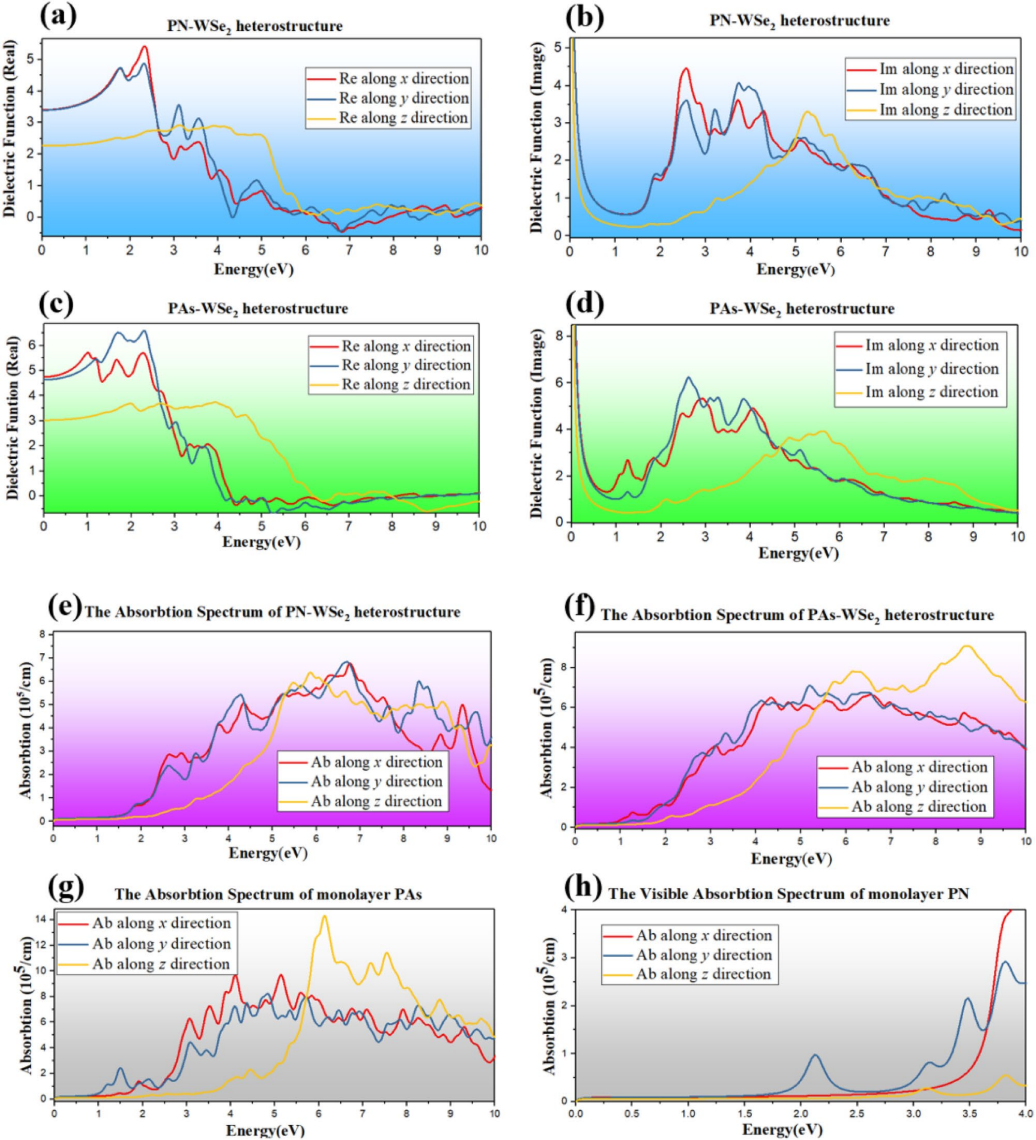
(**a**,**b**) The dielectric function’s real and imaginary part of PN-WSe_2_ vdW heterostructure; (**c**,**d**) The dielectric function’s real and imaginary part of PN-WSe_2_ heterostructure; (**e**,**f**) The absorption spectrum of PN/PAs-WSe_2_ heterostructure in 0–10 eV; (**g**) The absorption spectrum of monolayer PN in visible light region; (**h**) The absorption spectrum of monolayer PAs in 0–10 eV.

Many-body effect has a profound impact on the optical properties of 2D materials due to reduced coulomb screening. The Bethe–Salpeter approach can achieve a convincing agreement with experimental absorption spectra. In this work, instead, we introduce the so called scissors correction (SCI) to reduce the errors caused by the neglected many-body effects. The imaginary part of dielectric function of the PN/PAs-WSe_2_ vdW heterostructure along the y-direction are shown in Fig. S1 and Fig. S2. It indicates that the heterostructures have obvious exciton effect. However, our calculated results show that the shape of the spectrum based on IPA + scissors correction can reproduce that of GW + BSE. Considering the high computational cost of BSE, and also for comparison with the results reported PCE of other 2D heterostructures, the linear optical properties are discussed on basis of the independent-particle approximation (IPA) with scissors correction in present work. As shown in the Fig. 5a,c, the PN/PAs-WSe_2_ vdW heterostructures have the dielectric characteristic with high refractive index at low frequencies and the metallic characteristic due to negative refractive index at high frequencies. In particular, the dielectric functions for linear polarization along *x* and *y* direction can reach 5.42 and 6.58 at 2.33 eV, respectively. Although the dielectric functions along vertical polarization (*z*) are smaller than that along horizontal polarization (*x* and *y*), they also show strong response in the visible light region (0–5 eV). For the imaginary part, in case of horizontal polarization, PAs-WSe_2_ vdW heterostructures have three peaks at 2.57, 3.73 and 5.25 eV. There are three major peaks in 2.92, 2.62 and 5.65 eV for PN-WSe_2_ vdW heterostructure, which are shown in Fig. 5b,d. For the PN-WSe_2_ vdW heterostructures, there is a strong and wide absorption peak in visible region (350–682 nm) compared with component PN monolayer, especially for the z-direction components as shown in Fig. 5e, the absorption intensity of heterostructure is 30 times higher than that of PN monolayer in Fig. 5h. It could be interpreted that strong interlayer coupling leads to denser DOS for those heterostructures. It is clearly that the type II band structure of PN-WSe_2_ vdW heterostructure and large absorption coefficient in visible light region ensure its potential application for solar cells which be estimated quantitatively by the power conversion efficiency (PCE) formula given by Scharber et al.^25^ The upper limit for PCE can be described as:

$$\eta = \frac{{J_{sc} V_{oc} \beta_{FF} }}{{P_{solar} }} = \frac{{0.65\left( {E_{g}^{d} - \Delta E_{c} - 0.3} \right)\mathop \smallint \nolimits_{{E_{g}^{d} }}^{\infty } \frac{{J_{ph} \left( {\hbar \omega } \right)}}{\hbar \omega }d\left( {\hbar \omega } \right)}}{{\mathop \smallint \nolimits_{0}^{\infty } J_{ph} \left( {\hbar \omega } \right)d\left( {\hbar \omega } \right)}}$$

**Figure 6:**
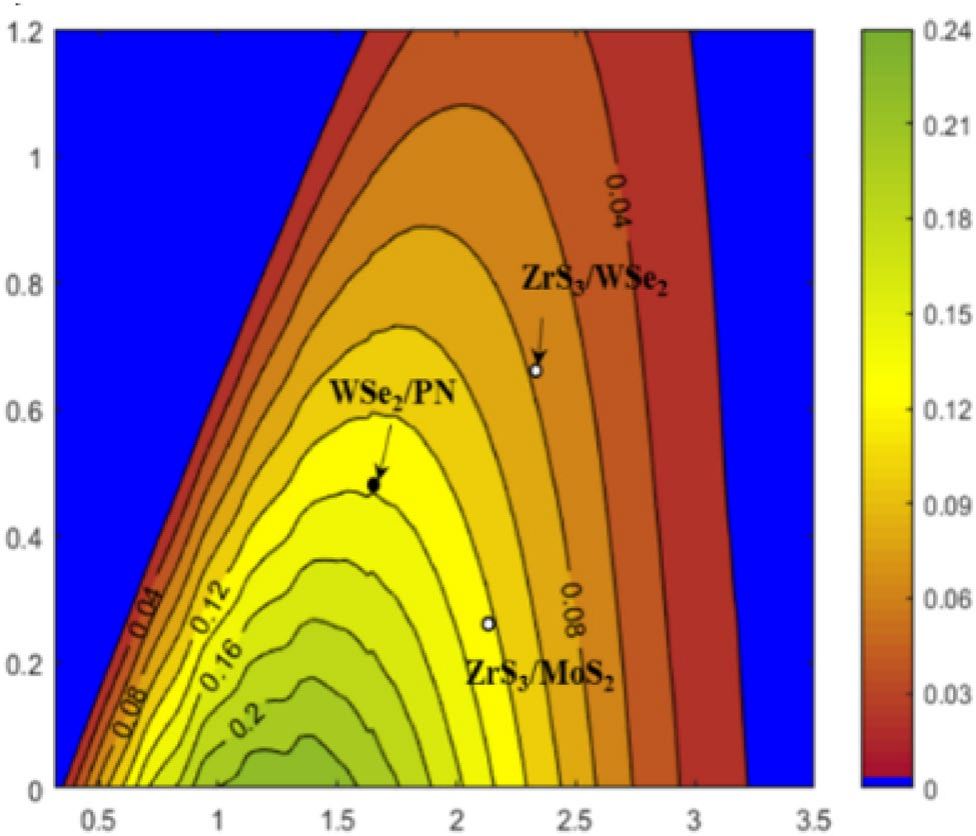
Power Conversion Efficiency contour computed plot. The black solid point marks the position of the type-II PN-WSe_2_ vdW heterostructure. The white hole shows the PCE for two novel heterostructures of ZrS3-MoS_2_ and ZrS_3_-WSe_2_^28^.

From the band alignment in Fig. 3a, the band gap of WSe_2_ donor is $E_{g}^{d} = 1.56\;{\text{eV}}$, the conduction band offset (CBO) for PN-WSe_2_ vdW heterostructure is $\Delta {\text{E}}_{{\text{c}}} = 0.47\; {\text{eV}}$, the band fill factor $\upbeta _{{{\text{FF}}}}$ is 0.65 and the 0.3 eV is the empirical estimation of kinetic energy conversion loss^25^. $J_{ph} \left( {\hbar \omega } \right)$ is AM1.5 solar energy flux (Wm^−2^ eV^−1^). As shown in Fig. 6, the solar power conversion efficiency contour at efficiency of 13.8%, PN-WSe_2_ vdW heterostructure possesses a high PCE at the level of CBO, higher than the organic solar cells(11.7%)^26^ vdW heterostructure, the MoS_2_/p-Si solar cells (5.23%)^27^ vdW heterostructure and the type-II GaTe-InSe (9.1%)^28^ which are recently reported. This is attributed to the reduced band gap of PN donor in contrast to that of MoS_2_ and reduced CBM differences between PN and WSe_2_ monolayers. The result illustrates a new approach for an efficient solar energy conversion on PN-WSe_2_ vdW heterostructure in 2D excitonic solar cells.

For PAs-WSe_2_ vdW heterostructure in Fig. 5f, it has a very strong absorption peak is at 8.62 eV with absorption coefficient 9.1 × 10^5^/cm and extremely wide absorption band in the ultraviolet region when it is polarized along vertical direction. Compared with the Janus b-PAs^29^, α-PAs has a larger direct optical band gap and relative high response at the peak area because of the different crystal structure. But when introducing it into heterostructure with WSe_2_, as shown in Fig. 5g, the high response occurs blue shift and shows high absorption characteristics in the ultraviolet region, which is more wide and gentle change before 10 eV. It implies that PAs-WSe_2_ vdW heterostructure is the potential candidate for highly sensitive UV detectors.

## Summary

In summary, we have systematically predicted the electronic and optical properties of PN/PAs (Black Phosphorus phase)-WSe_2_ vdW heterostructures with indirect and tunable band gaps and their modulations under the external electrical field. For the PAs-WSe_2_ vdW heterostructure, its VBM and CBM are both contributed by PAs monolayer, resulting in type-I band alignment. Whereas for the PN-WSe_2_ vdW heterostructure, its VBM and CBM results from WSe_2_ and PN monolayer respectively, which leads to a type-II band alignment indicating it is beneficial to the spatial separation of photogenerated electron–hole pairs. The external electrical field not only tunes both heterostructures’ band edges, contributors of VBM and CBM and band gaps, but also changes the Schottky barrier from *p*-type to *n*-type and turns the PAs-WSe_2_ vdW heterostructure into a type-II heterostructure from type-I. Furthermore, this research also predicts they have strong and considerable highly anisotropic optical response, which greatly enhance the optical performance of PN and PAs, enabling novel applications in optoelectronics from visible to ultraviolet regions. The extremely wide absorption spectrum in the ultraviolet region makes the PAs-WSe_2_ heterostructure is likely to become an alternative UV detector material. For the type-II PN-WSe_2_ heterostructure, it has a great visible light absorption and the high solar PCE at the level of CBO of 13.8%. It does show great application potential in exciton solar cells. Our results demonstrate that these two 2D-materials have great potential in the application of nano-optoelectronic devices based on van der Waals heterostructure.

## Supplementary information

Supplementary Information.

## Data Availability

The data that supports the findings are available upon request from the corresponding author.
