# Supplementary material for: PN/PAs-WSe2 van der Waals heterostructures for solar cell and photodetector
Source: Sci Rep. 2020 Oct 14;10:17213. doi: 10.1038/s41598-020-73152-7 (PMC7560845; doi:10.1038/s41598-020-73152-7)
Supplement: Supplementary file 1 — Supplementary Information. [file 41598_2020_73152_MOESM1_ESM.docx]

Supporting Information of the article “PN/PAs-WSe_2_ van der Waals heterostructures for solar cell and photodetector”

Xinyi Zheng^1^ , Yadong Wei^1^, Kaijuan Pang^1^, Ngeywo Tolbert Kaner^1^, Dalin Kong^1^, Xiaodong Xu^1^, Jianqun Yang^2^, Xingji Li^2*^, and Weiqi Li^1,3*^

^1^Harbin Institute of Technology, School of Physics, Harbin, 150001, China
^2^Harbin Institute of Technology, School of Materials Science and Engineering, Harbin, 150001, China
^3^Collaborative Innovation Center of Extreme Optics, Shanxi University, Taiyuan, 030006, China.
^*^tccliweiqi@hit.edu.cn


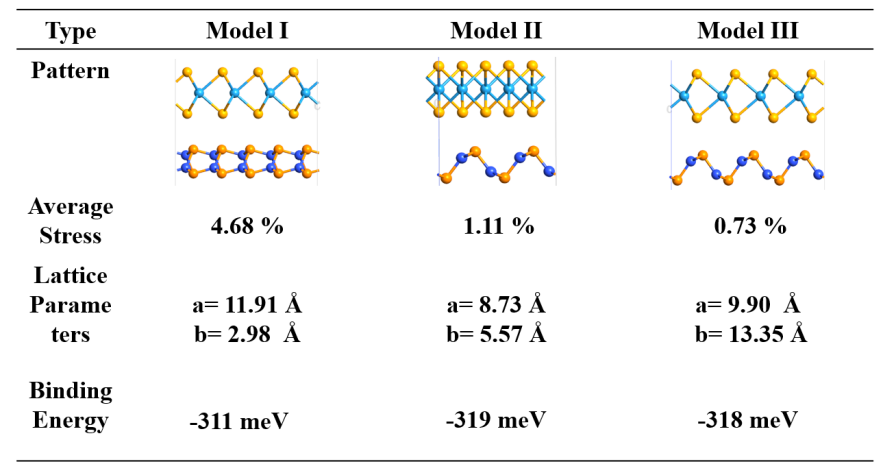


**Table.S1** Other different stacked modes of the PN-WSe_2_ vdW heterostructure’s

lattice parameter and binding energies.


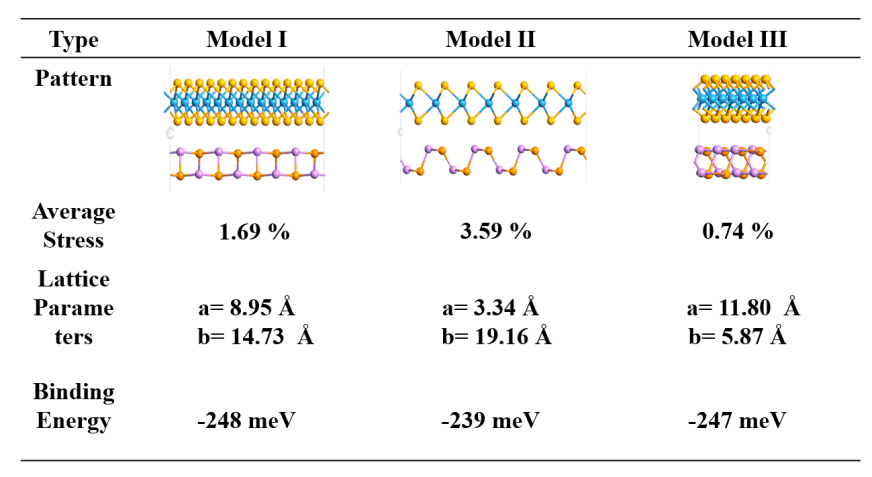


**Table.S2** Other different stacked modes of the PAs-WSe_2_ vdW heterostructure’s

lattice parameter and binding energies.


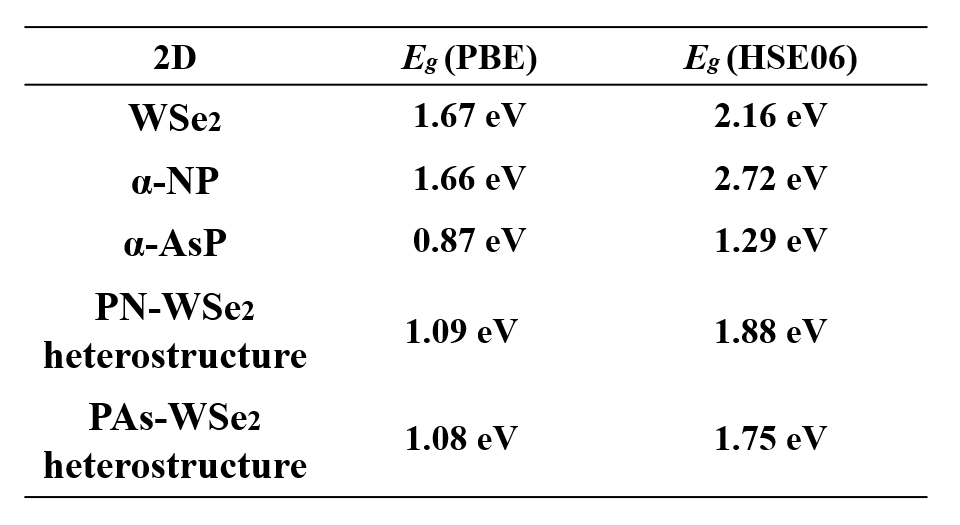


**Table.S3** The band gaps calculated by the DFT-PBE and HSE06 method.


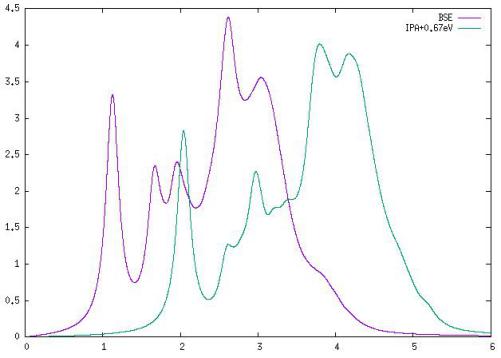


**Figure.S1** The imaginary part of the PAs-WSe_2_ vdW heterostructure’s dielectric function

excited along the y-axis obtained by G_0_W_0_ and BSE method .


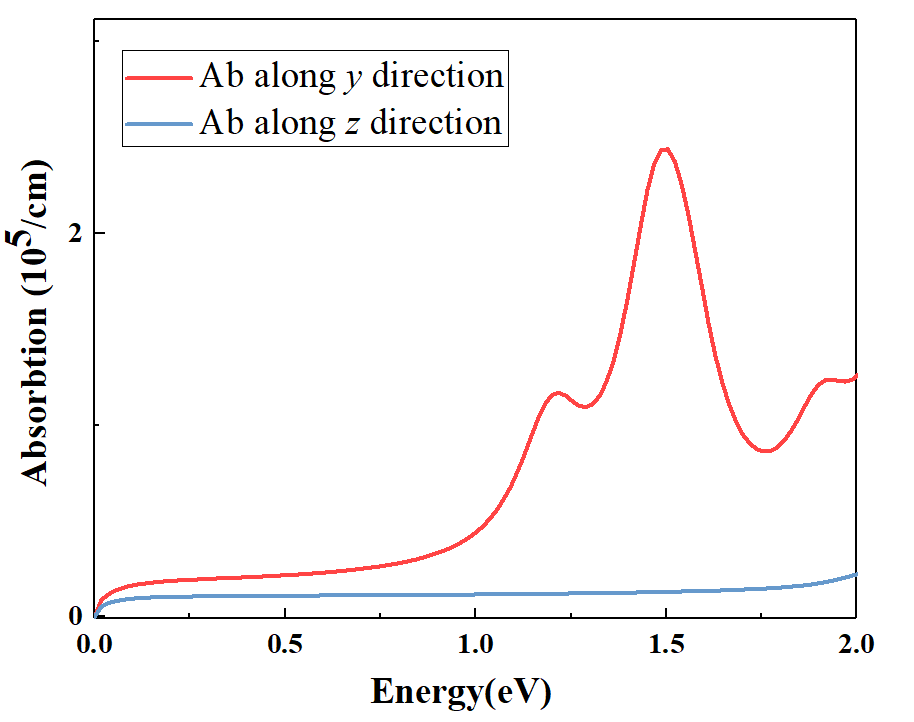


**Figure.S2** The Absorption Spectrum below 2 eV of monolayer PAs


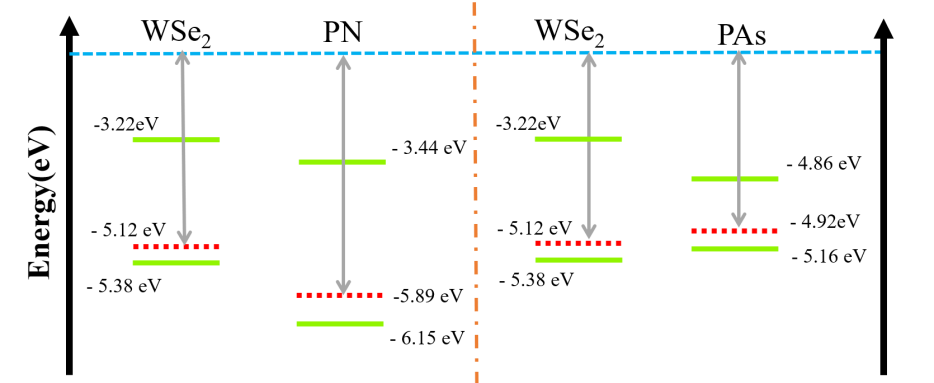


### **Figure S3.** The work function and CBM and VBM relative to the vacuum energy level of WSe_2_、PN、PAs calculated by HSE06 method for judging the band alignment.
